# Effect of the Modified Dietary Approach to Stop Hypertension (DASH) Diet on Glycemic Control and Insulin Resistance in Patients With Diabetes

**DOI:** 10.7759/cureus.88698

**Published:** 2025-07-24

**Authors:** Supriya Swami, L. H Ghotekar, Anupam Prakash, Ramesh Aggarwal, Shubhalaxmi Margekar, Parijat Gogoi, Apoorv Ojha

**Affiliations:** 1 Internal Medicine, Lady Hardinge Medical College, New Delhi, IND; 2 Biochemistry, Lady Hardinge Medical College, New Delhi, IND

**Keywords:** glycemic control, hba1c, homa-ir, insulin resistance, lifestyle intervention, modified dash diet, type 2 diabetes mellitus

## Abstract

Background: Type 2 diabetes mellitus (T2DM) is a chronic metabolic disease largely fueled by insulin resistance and defective insulin secretion. Nutritional modification is a central aspect of glycemic control and the prevention of diabetes-related complications. The Dietary Approach to Stop Hypertension (DASH) diet, initially designed to treat hypertension, has demonstrated encouraging evidence in enhancing metabolic control in T2DM when appropriately adapted. The aim of this study was to assess the impact of a modified DASH diet on insulin resistance and glycemic control among adults with T2DM.

Methods: This quasi-experimental non-randomized trial was conducted at Lady Hardinge Medical College and Associated Hospitals, New Delhi, from May 2023 to November 2024. Sixty patients with T2DM (glycated hemoglobin or HbA1c < 7.5%) were assigned to two groups: Group A (n = 30) received counseling on a modified DASH diet, and Group B (n = 30) was provided with a standard diabetic diet. Baseline and 12-week assessments consisted of fasting blood glucose (FBG), serum insulin, HbA1c, lipid profile, BMI, and homeostatic model assessment of insulin resistance (HOMA-IR). Dietary compliance was reinforced with regular follow-up.

Results: At baseline, the two groups were similar in age, BMI, and glycemic measures. Group A showed significantly greater decreases at 12 weeks in FBG (p = 0.001), HbA1c (within-group p < 0.001; between-group p = 0.057), and BMI (p < 0.001) compared to Group B. The reduction in HOMA-IR was also more significant in Group A, although intergroup changes at 12 weeks were significant at a borderline level but failed to meet the threshold for statistical significance. Generalized estimating equation analysis validated notable within-group changes in Group A in weight, BMI, and glycemic parameters.

Conclusion: The modified DASH diet demonstrated improved glycemic control, insulin resistance, and weight results at 12 weeks compared to a normal diabetic diet. These data attest to its utility as an effective dietary approach in the overall management of T2DM.

## Introduction

Type 2 diabetes mellitus (T2DM) is a long-term metabolic disorder defined by insulin resistance and defective insulin secretion, leading to sustained hyperglycemia. Chronic hyperglycemia, if left uncontrolled, can lead to long-term complications in critical organs such as the eyes, kidneys, heart, and nervous system [1,2]. T2DM in India is quickly on the rise, mainly contributed by lifestyle patterns such as unhealthy dietary habits and sedentary behavior [3-5]. As with its forward momentum and inherent complications, the effective management protocol is vital, with dietary modification central to its strategies [6,7].

T2DM development and disease progression have been established to be directly linked by multiple studies with regards to diet. Dietary patterns high in refined carbohydrates, saturated fats, and processed foods have been found to exacerbate insulin resistance, whereas those rich in whole grains, fruits, vegetables, lean proteins, and healthy fats are associated with improved glycemic control and enhanced insulin sensitivity [8,9]. These eating habits not only contribute to the control of blood glucose levels but also to the reduction of risk for related cardiovascular complications, a major cause of morbidity and mortality among patients with diabetes [9].

Randomized controlled trials and meta-analyses provide evidence for the efficacy of particular eating habits in the control of T2DM. The Mediterranean diet, which involves high consumption of fruits, vegetables, whole grains, olive oil, nuts, and fish and poultry in moderation, has shown considerable benefits. In the PREDIMED study, following the Mediterranean diet led to a 30% decrease in the risk of T2DM, along with changes in glycated hemoglobin (HbA1c) and lipid profiles [10,11]. Likewise, the Dietary Approach to Stop Hypertension (DASH) diet, which was initially formulated for the management of hypertension, is focused on consuming fruits, vegetables, low-fat dairy products, whole grains, and lean meats while restricting sodium and saturated fats. It has reflected remarkable improvement in glycemic control and insulin sensitivity in individuals with T2DM [12-14].

The DASH diet, while retaining its original principles, has been adapted in certain studies to improve its impact on glycemic control, and such modifications have demonstrated additional benefits. Research has demonstrated that it decreases fasting blood glucose (FBG), HbA1c, and insulin resistance considerably [15]. Additionally, the diet has a positive effect on cardiovascular health by lowering blood pressure and cholesterol levels, features that are particularly useful in patients with T2DM, who are at increased cardiovascular risk [16]. Its fiber content is also very high, which additionally helps with insulin sensitivity and weight control, both important aspects in diabetes management [17].

Combining diet with other lifestyle changes, such as physical activity and behavioral change, improves long-term results. The Look AHEAD (Action for Health in Diabetes) trial proved that diet and exercise together result in long-term weight loss, better glycemic control, and lower cardiovascular risk [13,14]. Though the advantages are apparent, translation to real-world practice can be difficult based on issues such as limited availability of healthy food, cost, and compliance.

Dietary change, particularly through evidence-based interventions such as the Mediterranean and modified DASH diets, is a core component of the prevention and control of T2DM. When blended with physical activity and behavioral therapy, these interventions have the potential to significantly enhance patient outcomes and quality of life. The study aims to find out the proportion of population showing decrease in insulin resistance and HbA1c after modified DASH diet.

## Materials and methods

This quasi-experimental non-randomized study was conducted at Lady Hardinge Medical College and Associated Hospitals, New Delhi, from May 2023 to November 2024. The study participants were adult patients (≥18 years) with T2DM, who attended the OPD of Lady Hardinge Medical College and Smt. Sucheta Kriplani Hospital, and had a baseline HbA1c <7.5%. Patients were excluded from the study if they were pregnant; had ongoing infections; had malignancy, nephropathy, or chronic kidney disease; were critically ill; or were already on a modified DASH diet.

Using Azadbakht et al. [18] and applying Snedecor and Cochran's formula, at least 30 patients were allotted to each group, with 99% power and 95% confidence. The patients who met the inclusion criteria were grouped into two sets: Group A (n = 30), who started on the modified DASH diet, and Group B (n = 30), who received a regular diabetic diet. The clinical evaluation comprised a thorough history, anthropometry (weight, height, and BMI), and general physical examination. Initial investigations included FBG, lipid profile, serum insulin, and HbA1c. Homeostatic model assessment of insulin resistance (HOMA-IR) was determined with the following formula: HOMA-IR = (Fasting insulin (µU/mL) × Fasting glucose (mg/dL))/405. Measurements were repeated at a three-month follow-up.

Vegetables, fruits, whole grains, low-fat dairy, lean meats, legumes, nuts, and restricted saturated fats (<10% of total calories), added sugars (<10% of total calories), sodium (<2300 mg/day), and alcohol (≤1 drink/day in women and ≤2 in men) were components of the modified DASH diet. Participants were followed up at four and eight weeks for reinforcement and dietary adherence. Follow-up assessments involved re-evaluation of BMI, insulin, HbA1c, lipid profile, and HOMA-IR. Patients were counseled and educated on healthy lifestyle practices during the study duration. Subgroup analysis was also performed to determine the effect of BMI on outcomes (<23 vs. ≥23 kg/m²).

Statistical analysis

Data were entered and coded in Microsoft Excel (Microsoft Corporation, Washington, United States) and then analyzed using SPSS Statistics for Windows, Version 23.0 (IBM Corp., Armonk, NY). Continuous variables were reported as means ± standard deviation or medians with interquartile ranges, and categorical variables as frequencies and percentages. Independent sample t-tests were employed to compare means between two groups, and one-way analysis of variance with Tukey's honestly significant difference test for more than two groups. Non-parametric counterparts (the Wilcoxon test and the Kruskal-Wallis test) were employed when the normality assumptions were not met. Chi-square test or Fisher's exact test (where appropriate) was applied for comparisons between categories. The correlation of continuous variables was tested using Pearson's or Spearman's correlation coefficients. p-value <0.05 was regarded as statistically significant.

## Results

The study recruited 60 participants divided equally into Group A (modified DASH diet) and Group B (usual diabetic diet). The mean age was 53.33 ± 8.98 years, with 46.7% of the participants in the age group of 51-60 years. Females outnumbered males (65%), and the mean duration of diabetes was 4.07 ± 2.54 years, with 61.7% of them being diabetic for 1-5 years. All continuous variables such as age and diabetes duration were normally distributed, as also verified by the skewness and Shapiro-Wilk test, thus ensuring the suitability of parametric tests for statistical analysis (Table 1).

**Table 1:** Baseline characteristics of study participants (N = 60) DASH: Dietary Approach to Stop Hypertension

Parameter	Category	Frequency (n)	Percentage (%)	95% Confidence Interval
Age (Years)	31–40	6	10.0	–
	41–50	15	25.0	–
	51–60	28	46.7	–
	61–70	9	15.0	–
	71–80	2	3.3	–
Gender	Male	21	35.0	23.4%–48.5%
	Female	39	65.0	51.5%–76.6%
Duration of Diabetes	<1 Year	6	10.0	4.1%–21.2%
	1–5 Years	37	61.7	48.2%–73.6%
	6–10 Years	17	28.3	17.8%–41.6%
Group Allocation	Group A (Modified DASH Diet)	30	50.0	37.7%–62.3%
	Group B (Diabetic Diet)	30	50.0	37.7%–62.3%

There was no significant statistical difference in age or BMI (both baseline and 12 weeks) between Group A (modified DASH diet) and Group B (diabetic diet). Significant decreases were noted for FBG at baseline (p = 0.046) and 12 weeks (p = 0.001) in favor of Group A, showing improved glycemic control with the modified DASH diet. Though levels of HbA1c in Group A declined compared with Group B after 12 weeks, the disparity was narrowly failing to achieve statistical significance (p = 0.057) and indicated a possible benefit that may become significant using a higher patient number or more extended follow-up (Table 2).

**Table 2:** Comparison of clinical parameters between groups BMI: Body mass index; DASH: Dietary Approach to Stop Hypertension; FBG: Fasting blood glucose; HbA1c: Glycated hemoglobin

Parameter	Group A (DASH Diet)	Group B (Diabetic Diet)	Test Used	Test Statistic (t/U)	p-Value
Age (Years)	52.10 ± 9.65; 52.5 (47.25–56)	54.57 ± 8.24; 55 (48–59.5)	Independent t-Test	t = -1.065	0.291
BMI (Baseline) (kg/m²)	29.68 ± 6.13; 29.52 (22.62–33.94)	27.86 ± 5.56; 27.75 (21.52–31.92)	Mann–Whitney U Test	U = 551.000	0.137
BMI (12 Weeks)	28.40 ± 5.36; 28.12 (23.36–31.54)	27.71 ± 5.32; 29.03 (22.01–31.93)	Mann–Whitney U Test	U = 484.500	0.615
FBG (Baseline) (mg/dL)	124.03 ± 17.17; 124 (110.5–137)	132.87 ± 16.38; 133.5 (123–142.75)	Independent t-Test	t = -2.039	0.046
FBG (12 Weeks) (mg/dL)	108.77 ± 10.71; 108 (100.25–112)	120.57 ± 14.09; 120 (112–127)	Mann–Whitney U Test	U = 224.000	0.001
HbA1c (Baseline) (%)	6.86 ± 0.47; 6.95 (6.65–7.2)	6.72 ± 0.51; 6.8 (6.32–7.1)	Mann–Whitney U Test	U = 521.500	0.292
HbA1c (12 Weeks) (%)	6.50 ± 0.59; 6.6 (6.1–6.88)	6.75 ± 0.56; 7 (6.32–7.1)	Mann–Whitney U Test	U = 321.000	0.057

During the 12-week duration, Group A (modified DASH diet) showed considerable decreases in weight and BMI, both within the group and compared to Group B (diabetic diet), as indicated by the p-values of the generalized estimating equation analysis (p < 0.001 for both parameters). FBG values were considerably reduced within both groups throughout the period, with Group A indicating improved control at the 12-week interval (p = 0.001); however, the difference between the change within and between groups throughout was not statistically significant (p = 0.458). For HbA1c, Group A demonstrated a statistically significant decrease within the group (p < 0.001), and the total change was also significantly larger than Group B (p < 0.001), although the difference between groups at 12 weeks was non-significantly marginal (p = 0.057). These results indicate that the modified DASH diet was superior in enhancing glycemic control and weight outcomes at 12 weeks than the standard diabetic diet (Table 3).

**Table 3:** Comparison of change over time between groups BMI: Body mass index; DASH: Dietary Approach to Stop Hypertension; FBG: Fasting blood glucose; GEE: Generalized estimating equation; HbA1c: Glycated hemoglobin; SD: Standard deviation

Parameter	Timepoint	Group A (DASH Diet) (Mean ± SD)	Group B (Diabetic Diet) (Mean ± SD)	p-Value (Between Groups at Timepoint)	Test Statistic (t/U)	p-Value (Within Group)	Overall p-Value (GEE)
Weight (kg)	Baseline	77.37 ± 13.65	77.87 ± 15.16	0.894	t = -0.134	A: <0.001, B: 0.389	<0.001
	12 Weeks	74.10 ± 11.85	77.43 ± 14.40	0.332	t = -0.975		
BMI (kg/m²)	Baseline	29.68 ± 6.13	27.86 ± 5.56	0.137	U = 551.000	A: <0.001, B: 0.393	<0.001
	12 Weeks	28.40 ± 5.36	27.71 ± 5.32	0.615	U = 484.500		
FBG (mg/dL)	Baseline	124.03 ± 17.17	132.87 ± 16.38	0.046	t = -2.039	A: <0.001, B: <0.001	0.458
	12 Weeks	108.77 ± 10.71	120.57 ± 14.09	0.001	U = 224.000		
HbA1c (%)	Baseline	6.86 ± 0.47	6.72 ± 0.51	0.292	U = 521.500	A: <0.001, B: 0.706	<0.001
	12 Weeks	6.50 ± 0.59	6.75 ± 0.56	0.057	U = 321.000		

The study found that at baseline, the two groups had similar metabolic parameters such as HOMA-IR, triglyceride to high-density lipoprotein cholesterol (TG/HDL-C) ratio, and high-density lipoprotein (HDL) level. But at 12 weeks, Group A (modified DASH diet) demonstrated a much better improvement in insulin sensitivity (HOMA-IR: p = 0.002) and a more significant decrease in the TG/HDL-C ratio (p < 0.001), representing better lipid profile improvement than Group B (diabetic diet). Although low-density lipoprotein (LDL) levels were lower in Group B at baseline (p = 0.028), no significant difference was observed in triglyceride (TG) levels. These results indicate that the modified DASH diet was superior in enhancing insulin resistance and lipid markers, thereby pointing toward its use as a superior therapeutic regimen for metabolic health management (Table 4).

**Table 4:** Association between groups and metabolic parameters HDL: High-density lipoprotein; HOMA-IR: Homeostatic model assessment of insulin resistance; IQR: Interquartile range; LDL: Low-density lipoprotein; SD: Standard deviation; TG: Triglyceride; TG/HDL-C: Triglyceride to high-density lipoprotein cholesterol

Parameter	Timepoint	Group A (Mean ± SD)	Group B (Mean ± SD)	Median (IQR) – A	Median (IQR) – B	p-Value (Between Groups)	Test Statistic (U/t)	Test Used
HOMA-IR	Baseline	3.03 ± 1.15	3.60 ± 1.50	2.86 (2.32–3.59)	2.99 (2.53–4.68)	0.219	U ≈ 540.0	Wilcoxon-Mann-Whitney Test
	12 Weeks	2.32 ± 0.93	2.94 ± 0.91	2.13 (1.87–2.72)	2.73 (2.24–3.52)	0.002	U ≈ 290.0	Wilcoxon-Mann-Whitney Test
TG/HDL-C Ratio	Baseline	3.81 ± 1.22	3.51 ± 1.01	3.58 (3.07–4.30)	3.40 (2.75–4.00)	0.348	U ≈ 520.0	Wilcoxon-Mann-Whitney Test
	12 Weeks	2.55 ± 1.01	3.17 ± 0.76	2.54 (2.03–2.81)	3.02 (2.68–3.47)	<0.001	U ≈ 230.0	Wilcoxon-Mann-Whitney Test
HDL (mg/dL)	Baseline	46.47 ± 7.87	47.19 ± 9.48	47 (40.25–52)	47 (39.42–54.25)	0.749	t ≈ -0.32	Independent t-Test
TG (mg/dL)	Baseline	172.10 ± 44.84	158.47 ± 24.06	166.5 (140–195.5)	163 (140–177)	0.258	U ≈ 500.0	Wilcoxon-Mann-Whitney Test
LDL (mg/dL)	Baseline	120.33 ± 29.07	106.27 ± 17.75	113.5 (102.25–132.75)	102.5 (92.25–120.25)	0.028	t ≈ 2.27	Independent t-Test

Both groups, A and B, had similar HOMA-IR, HDL, and TG levels at baseline. At 12 weeks, Group A had a greater decrease in HOMA-IR (p = 0.002) and a greater increase in HDL levels (p = 0.023) than Group B, suggesting improved insulin sensitivity and lipid profile in Group A. Both groups had significant changes within themselves regarding HOMA-IR and TG levels (p < 0.001), whereas Group A had a better change in TGs over time (p < 0.001). Overall, the differences between these metabolic markers in the groups were not appreciably different in HOMA-IR (p = 0.861), yet Group A had more favorable results in HDL and TG than Group B (Table 5).

**Table 5:** Comparison of change in HOMA-IR, HDL, and TG over time between Groups A and B HDL: High-density lipoprotein; HOMA-IR: Homeostatic model assessment of insulin resistance; SD: Standard deviation; TG: Triglyceride; t: Student’s t-test; U: Mann–Whitney U test; W: Wilcoxon signed-rank test; χ²: Chi-square test

Parameter	Group A (Mean ± SD)	Group B (Mean ± SD)	p-Value (Between Groups at Timepoint)	Test Statistic (Between Groups)	p-Value (Within Group Change Over Time)	Test Statistic (Within Group)	p-Value (Between Groups Change Over Time)	Test Statistic (Change Over Time Between Groups)
HOMA-IR (Baseline)	3.03 ± 1.15	3.60 ± 1.50	0.219	U ≈ 540	<0.001	W ≈ 150	0.861	χ² ≈ 0.03
HOMA-IR (12 Weeks)	2.32 ± 0.93	2.94 ± 0.91	0.002	U ≈ 290	<0.001	W ≈ 120	0.861	χ² ≈ 0.03
HDL (Baseline)	46.47 ± 7.87	47.19 ± 9.48	0.749	t ≈ -0.32	0.002	t ≈ 3.20	0.008	χ² ≈ 7.0
HDL (12 Weeks)	53.17 ± 10.68	47.60 ± 7.37	0.023	t ≈ 2.40	0.773	t ≈ 0.30	0.008	χ² ≈ 7.0
TG (Baseline)	172.10 ± 44.84	158.47 ± 24.06	0.258	U ≈ 500	<0.001	W ≈ 130	<0.001	χ² ≈ 11.0
TG (12 Weeks)	128.80 ± 32.88	147.57 ± 24.22	0.003	U ≈ 320	0.043	W ≈ 170	<0.001	χ² ≈ 11.0

The comparison between individuals with BMI <23 kg/m² and those with BMI ≥23 kg/m² in the modified DASH diet group indicates a number of important differences. For example, participants with increased BMI had substantially higher baseline and 12-week body weight and BMIs (p < 0.001), as expected. Also, the decrease in BMI after 12 weeks was greater in the ≥23 kg/m² group (-1.77 vs. -0.12 kg/m²; p = 0.001), suggesting a more significant effect of the diet on weight reduction in the overweight/obese population. A notable difference was also noted in LDL reduction, which was greater in the lower BMI group (-52.67 vs. -22.38 mg/dL; p = 0.039), proposing a beneficial lipid-lowering effect in lean subjects. Other parameters such as age, gender distribution, glycemic measures (HbA1c and HOMA-IR), and changes in lipid profiles (HDL, TG, and TG/HDL-C) were not statistically different between groups, but trends toward greater improvement in HDL were seen more clearly among those with increased BMI. As a whole, the modified DASH diet produced differential effects across baseline BMI with greater weight loss and BMI loss in the overweight group and increased LDL reduction among lean participants (Table 6).

**Table 6:** Comparison of parameters between BMI <23 kg/m² and ≥23 kg/m² in the modified DASH diet group BMI, Body mass index; HbA1c, Glycated hemoglobin; HDL, High-density lipoprotein; HOMA-IR, Homeostatic model assessment of insulin resistance; LDL, Low-density lipoprotein; SD, Standard deviation; TG, Triglyceride; TG/HDL-C, Triglyceride to high-density lipoprotein cholesterol ***indicates p < 0.05.

Parameters	BMI <23 kg/m² (n = 9)	BMI ≥23 kg/m² (n = 21)	p-Value	Test Statistic (U or Fisher's exact)	Test Used
Age (Years)	48.67 ± 12.09	53.57 ± 8.30	0.134	U ≈ 61.0	Wilcoxon-Mann-Whitney Test
Gender			0.681	p = 0.681	Fisher's Exact Test
Male	2 (22.2%)	7 (33.3%)			
Female	7 (77.8%)	14 (66.7%)			
Duration of Diabetes (Years)	4.23 ± 2.80	4.72 ± 2.66	0.632	U ≈ 75.5	Wilcoxon-Mann-Whitney Test
Weight (kg) (Baseline)***	61.44 ± 4.00	84.19 ± 10.04	<0.001	U ≈ 0.0	Wilcoxon-Mann-Whitney Test
Weight (kg) (12 Weeks)***	61.11 ± 5.58	79.67 ± 9.09	<0.001	U ≈ 0.0	Wilcoxon-Mann-Whitney Test
BMI (kg/m²) (Baseline)***	22.07 ± 0.55	32.94 ± 4.13	<0.001	U ≈ 0.0	Wilcoxon-Mann-Whitney Test
BMI (kg/m²) (12 Weeks)***	21.94 ± 1.41	31.17 ± 3.75	<0.001	U ≈ 0.0	Wilcoxon-Mann-Whitney Test
HbA1c (%) (Baseline)	6.74 ± 0.49	6.91 ± 0.46	0.440	U ≈ 79.0	Wilcoxon-Mann-Whitney Test
HbA1c (%) (12 Weeks)	6.52 ± 0.51	6.50 ± 0.63	0.964	U ≈ 94.0	Wilcoxon-Mann-Whitney Test
HOMA-IR (Baseline)	2.92 ± 1.18	3.08 ± 1.16	0.657	U ≈ 77.5	Wilcoxon-Mann-Whitney Test
HOMA-IR (12 Weeks)	2.63 ± 1.37	2.19 ± 0.66	0.824	U ≈ 74.0	Wilcoxon-Mann-Whitney Test
HDL (mg/dL) (Baseline)	45.11 ± 6.51	47.05 ± 8.46	0.570	U ≈ 78.0	Wilcoxon-Mann-Whitney Test
HDL (mg/dL) (12 Weeks)	47.78 ± 10.02	55.48 ± 10.32	0.085	U ≈ 53.5	Wilcoxon-Mann-Whitney Test
TG (mg/dL) (Baseline)	175.44 ± 31.02	170.67 ± 50.23	0.483	U ≈ 79.5	Wilcoxon-Mann-Whitney Test
TG (mg/dL) (12 Weeks)	128.89 ± 55.62	128.76 ± 18.17	0.651	U ≈ 84.0	Wilcoxon-Mann-Whitney Test
LDL (mg/dL) (Baseline)	129.89 ± 32.41	116.24 ± 27.33	0.389	U ≈ 75.0	Wilcoxon-Mann-Whitney Test
LDL (mg/dL) (12 Weeks)	77.22 ± 14.20	93.86 ± 25.55	0.141	U ≈ 62.0	Wilcoxon-Mann-Whitney Test
TG/HDL-C (Baseline)	3.90 ± 0.48	3.77 ± 1.44	0.226	U ≈ 68.5	Wilcoxon-Mann-Whitney Test
TG/HDL-C (12 Weeks)	2.89 ± 1.63	2.40 ± 0.58	0.372	U ≈ 81.5	Wilcoxon-Mann-Whitney Test
Change in BMI (kg/m²) (12 Weeks)***	-0.12 ± 1.15	-1.77 ± 0.98	0.001	U ≈ 20.0	Wilcoxon-Mann-Whitney Test
Change in HbA1c (%) (12 Weeks)	-0.22 ± 0.31	-0.41 ± 0.50	0.339	U ≈ 66.0	Wilcoxon-Mann-Whitney Test
Change in HOMA-IR (12 Weeks)	-0.29 ± 1.89	-0.89 ± 0.67	0.263	U ≈ 68.0	Wilcoxon-Mann-Whitney Test
Change in HDL (mg/dL) (12 Weeks)	2.67 ± 11.88	8.43 ± 9.90	0.329	U ≈ 70.5	Wilcoxon-Mann-Whitney Test
Change in TG (mg/dL) (12 Weeks)	-46.56 ± 48.98	-41.90 ± 44.33	0.483	U ≈ 79.5	Wilcoxon-Mann-Whitney Test
Change in LDL (mg/dL) (12 Weeks)***	-52.67 ± 38.53	-22.38 ± 23.59	0.039	U ≈ 35.0	Wilcoxon-Mann-Whitney Test
Change in TG/HDL-C (12 Weeks)	-1.00 ± 1.44	-1.37 ± 1.25	0.657	U ≈ 78.0	Wilcoxon-Mann-Whitney Test

## Discussion

T2DM is a chronic metabolic disorder characterized by insulin resistance and impaired pancreatic β-cell function, culminating in persistent hyperglycemia. A substantial body of evidence underscores the critical role of lifestyle interventions in the comprehensive management of T2DM. Modified DASH diet provides an effective alternative in the management of diabetes mellitus. It is simple and easy to follow. Our study aimed to find out the impact of the modified DASH diet on glycemic control, insulin resistance, and change in the lipid profile.

In our study, the majority of participants fall within the 51-60 years age group, accounting for 46.7% of the total. The second-highest representation is from the 41-50 years group, constituting 25.0%. The data skew toward middle-aged and older adults, particularly those between 51 and 60 years, who are at a higher risk for T2DM. Our study's focus on this demographic aligns with the higher prevalence of T2DM in individuals above 40 years of age, highlighting the relevance of dietary interventions for this age group. According to the study conducted by Pradeepa and Mohan, the most affected age group typically spans from 40 to 60 years, aligning with findings from multiple population-based studies [19]. Participants were equally distributed into two groups, and there was no statistical difference in the ages of both groups. The majority of the sample population is female, making up 65.0% of the participants, while males constitute 35.0%.

The analysis of the weight data indicates that there was no statistically significant difference in weight between both groups at baseline (p = 0.894). The overall comparison of weight change between the two groups was statistically significant (p < 0.001). Participants in the modified DASH diet group experienced a statistically significant reduction in BMI from 29.68 ± 6.13 to 28.40 ± 5.36 over 12 weeks (p < 0.001), and body weight reduced from 77.37 ± 13.65 to 74.10 ± 11.85 kg. A study by Sandouk and Lansang [20] found that sustained weight loss of as little as 5% of initial body weight significantly improves glycemic control, lipid profiles, and blood pressure in individuals with diabetes and obesity.

The ADVANCE trial [21] analyzed weight changes and their predictors among 11,140 patients with type 2 diabetes over a median follow-up of five years. On average, participants gained 0.5 kg, though there was significant variability, with 25% gaining more than 2.6 kg and another 25% losing more than 1.8 kg. Younger age, higher baseline HbA1c levels, and the use of insulin or sulfonylureas were associated with greater weight gain, while metformin use and higher physical activity levels were linked to weight loss.

Group A had a higher average BMI (29.68 ± 6.13) compared to Group B (27.86 ± 5.56), but this difference was not statistically significant (p = 0.137). After 12 weeks, Group A's average BMI decreased significantly to 28.40 ± 5.36, while Group B's average BMI remained relatively unchanged at 27.71 ± 5.32. When comparing the overall change in BMI over time between the two groups, the results showed a statistically significant difference (p <0.001), indicating that Group A experienced a greater reduction in BMI compared to Group B over the 12-week period. The Look AHEAD trial reported an average BMI reduction of 1.7 kg/m² over one year with lifestyle interventions, while our study shows a reduction of 1.28 kg/m² in 12 weeks, indicating a faster response [14]. A meta-analysis by Nordmann et al. found that the Mediterranean diet led to a BMI decrease of 0.6 kg/m² over two years [22].

Group A had a slightly higher average HbA1c (6.86 ± 0.47%) compared to Group B (6.72 ± 0.51%), but this difference was not statistically significant (p = 0.292). After 12 weeks, Group A's average HbA1c significantly decreased to 6.50 ± 0.59%, while Group B's HbA1c remained stable at 6.75 ± 0.56%. The difference between the groups at 12 weeks approached significance (p = 0.057). Within the group, analysis showed that Group A experienced a statistically significant reduction in HbA1c over time (p < 0.001). In the Diabetes Prevention Program (DPP), lifestyle interventions, including dietary changes, reduced HbA1c by approximately 0.5% over one year, aligning with our findings of a 0.36% reduction in 12 weeks. The DPP Outcomes Study reported a 58% reduction in T2DM incidence with lifestyle interventions sustained over three years [23].

At baseline, Group B had a higher average HOMA-IR (3.60 ± 1.50) compared to Group A (3.03 ± 1.15), but this difference was not statistically significant (p = 0.219). After 12 weeks, both groups showed reductions in HOMA-IR. Group A’s HOMA-IR decreased significantly to 2.32 ± 0.93, while Group B’s HOMA-IR decreased to 2.94 ± 0.91. At 12 weeks, the difference between the groups became statistically significant (p = 0.002). HOMA-IR levels in the modified DASH diet group improved significantly, from 3.03 ± 1.15 at baseline to 2.32 ± 0.93 after 12 weeks (p < 0.001).

At baseline, Group A had a slightly higher TG/HDL-C ratio (3.81 ± 1.22) compared to Group B (3.51 ± 1.01), but the difference was not statistically significant (p = 0.348). After 12 weeks, both groups showed reductions in their TG/HDL ratios. Group A’s ratio decreased significantly to 2.55 ± 1.01, while Group B’s ratio also decreased, but to a lesser extent, at 3.17 ± 0.76. At this time point, the difference between the groups was statistically significant (p < 0.001). The Da Qing Diabetes Prevention Outcome Study followed 576 participants with impaired glucose tolerance for over 30 years, with 405 in the intervention group (lifestyle modification) and 135 in the control group [24]. The intervention group experienced a median delay in diabetes onset of 3.96 years compared to the control group (95% CI: 1.25-6.67; p = 0.0042). Additionally, the intervention group had a 26% reduction in cardiovascular disease events (hazard ratio: 0.74; 95% CI: 0.59-0.92; p = 0.0060) and a 35% reduction in microvascular complications (hazard ratio: 0.65; 95% CI: 0.45-0.95; p = 0.025). Cardiovascular disease deaths were reduced by 33% in the intervention group (hazard ratio: 0.67; 95% CI: 0.48-0.94; p = 0.022), and all-cause mortality was reduced by 26% (hazard ratio: 0.74; 95% CI: 0.61-0.89; p = 0.0015). Furthermore, the intervention group had an average increase in life expectancy of 1.44 years compared to the control group (95% CI: 0.20-2.68; p = 0.023). These results highlight the significant long-term benefits of lifestyle interventions in reducing diabetes onset, its complications, and mortality.

In our study, over 12 weeks, both groups showed significant lipid profile improvements, with Group A exhibiting greater reductions in LDL and TG and a significant increase in HDL compared to Group B. Group A's LDL dropped from 120.33 to 88.87 mg/dL and HDL rose to 53.17 mg/dL, while TGs decreased notably. Participants with a BMI ≥23 kg/m² had greater weight and BMI reductions, whereas those with a BMI <23 kg/m² showed a more pronounced LDL reduction. These results suggest that the modified DASH diet is effective, especially in improving lipid profiles and weight in higher BMI individuals and lowering LDL in lower BMI individuals.

In a meta-analysis by Nordmann et al. [22], the Mediterranean diet achieved similar reductions in LDL cholesterol (mean difference: -7.4 mg/dL) and TGs, with a modest increase in HDL cholesterol over two years. Sacks et al. [25] found that the DASH diet reduced LDL cholesterol by 10%-15%, but our study observed 26.2% reduction.

Limitations

This study’s 12-week duration limits insight into the long-term effects of the modified DASH diet. Diet adherence was self-reported without objective measures, which may introduce reporting bias and affect outcome validity. Blinding of participants and assessors was not feasible, potentially leading to bias. Physical activity, a key confounding factor in weight and insulin resistance outcomes, was not measured or controlled for in the analysis. Additionally, baseline differences between groups may have influenced the results. Finally, as the study was conducted in an urban northern Indian population, the findings may not be generalizable to other regions, ethnicities, or settings with different dietary habits, genetics, or healthcare access. Further research incorporating longer durations, objective adherence measures, physical activity assessment, and diverse populations is recommended.

## Conclusions

This study states that the modified DASH diet is a very efficient dietary strategy for controlling T2DM, effectively improving the control of blood glucose, insulin resistance, BMI, lipid profiles, and cardiovascular disease risk factors. Through the promotion of nutrient-dense foods and restriction of harmful food components, the modified DASH diet treats central to metabolic disturbances characteristic of T2DM. Its incorporation into everyday care, augmented by individualized nutritional advice and organized education, can improve adherence and long-term results. Though these results enhance the diet's therapeutic potential, additional longitudinal and population-diverse research will be required to establish its long-term effectiveness and applicability in different settings.
